# Knowledge, attitude and practice of physiotherapists towards promotion of physically active lifestyles in patient management

**DOI:** 10.1186/1472-6963-13-21

**Published:** 2013-01-14

**Authors:** Happiness A Aweto, Cynthia N Oligbo, Oluseun A Fapojuwo, Olajide A Olawale

**Affiliations:** 1 Department of Physiotherapy, College of Medicine, University of Lagos, Idi-Araba, Lagos, Nigeria

**Keywords:** Knowledge, Attitude, Practice, Physical activity

## Abstract

**Background:**

Physiotherapists as primary health care practitioners are well placed in promoting physically active lifestyles, but their role and practice towards its promotion among patients in Nigeria has not been fully investigated. This study was therefore aimed at determining the knowledge, attitude and practice of Nigerian physiotherapists towards promotion of non-treatment physical activity among patients.

**Methods:**

Three hundred and eight practicing physiotherapists from various public and private hospitals in 14 states of Nigeria completed an adopted 20-item questionnaire, which collected information on physical activity promotion in physiotherapy practice.

**Result:**

Respondents with good knowledge and attitude towards physical activity promotion in patient management were 196(63.6%) and 292(94.8%) respectively. Only 111 (36%) of the respondents counselled more than 10 patients in the past one month on the benefits of adopting a more physically active lifestyle. Chi-square analysis showed a significant association between low practice of physical activity promotion in patient management with inadequate consultation time (ℵ^2^ = 3.36, p = 0.043), years of working experience of physiotherapists (ℵ^2^ = 11.37, p =0.023) and relative physical activity levels of physiotherapists (ℵ^2^ = 11.82, p = 0.037). The need for Physical activity recommendation guideline was supported by 287 (97%) respondents.

**Conclusion:**

Nigerian physiotherapists have good knowledge and attitude towards promotion of physically active lifestyle in their patients but do not counsel many of them, due to insufficient consultation time. Integrating brief counselling into usual treatment sessions is perceived as the most feasible form of physical activity promotion in patient management.

## Background

In recent decades, physical inactivity has been linked with the onset of Non-Communicable Diseases (NCDs) and risk factors such as obesity, heart disease and cancer [1]. Global estimates by the World Health Organization have indicated that 10-16% of cases of breast, colon and rectal cancers, diabetes mellitus, and 22% of ischaemic heart diseases are caused by physical inactivity. Overall, 1.9 million deaths are attributable to physical inactivity [2]. Physical activity is a first-line therapy and protects against many chronic health conditions by improving glucose uptake, enhancing insulin sensitivity, improving blood lipid profiles, lowering blood pressure, improving the health of blood vessels, and protecting against obesity [3]. A physically active lifestyle has been shown to significantly reduce the risk of developing cardiovascular disease, obesity, type 2 diabetes mellitus, several forms of cancer and depression, strengthen bones and muscles, stabilize mental health and mood, increases one’s ability to perform daily activities and prevent falls [4]. Regular physical activity decreases all-cause mortality risk by 20% to 30% compared with insufficient activity [5].

Epidemiological studies have indicated that physical activity is accepted worldwide as a public health priority [4,6]. This epidemiological evidence has been synthesized into recommended levels of physical activity for metabolic health and cardiovascular disease prevention by the American College of Sports Medicine and the American Heart Association [7]. The recommendation states that adults should be active 5 days per week and have at least 30 minutes of moderate-intensity activity daily [7].

Primary health care practitioners are ideally positioned to promote physical activity as a health promotion measure. Many patients that attend primary care centres have health problems that could be prevented by a physically active lifestyle [8]. For the past decade, there has been a focus on using primary care physicians to promote physical activity. Strategies implemented by physicians have demonstrated mixed success [9] with most programmes showing modest effect only in the short term [10].

Physiotherapists have great potential for physical activity promotion [11]. They prescribe exercises for a wide range of conditions (mostly musculoskeletal) requiring rehabilitation. Currently, physiotherapy is mainly a tertiary prevention discipline, but equipped with the ideal skills and potential to act in a primary prevention role [12]. A survey of physiotherapists in the United States of America indicated that physical activity was the most frequent area of focus for health promotion behaviour [12].

Proficiency of prescription of non-treatment physical activity programmes during consultation can best be predicted by the physiotherapist’s level of confidence in applying such programmes in patient management [12]. Although it is believed that physiotherapists should be involved in physical activity promotion, the views of individual physiotherapists about their potential role in physical activity promotion practice or their confidence in engaging in such activities are not well known [11]. Hence, this study was designed to investigate the level of knowledge, attitude and counselling practice of physiotherapists towards promotion of non-treatment physical activity among patients.

## Methods

A total of three hundred and eight (308) practicing physiotherapists (166 males and 142 females) aged 20 years and above participated in this study. Physiotherapists were selected from various public and private hospitals in 14 states of Nigeria. The states were purposively selected based on relatively higher numbers of practicing physiotherapists in those states, as obtained from the Medical Rehabilitation Therapists Board of Nigeria.

Ethical approval was obtained from the Research and Ethics Committee of the Lagos University Teaching Hospital, Idi-Araba, Lagos. An informed consent was attached to a structured 20-item questionnaire which each of the participants completed.

### Questionnaire design

This questionnaire titled ‘Physical activity in physiotherapy practice Questionnaire’ was adopted from a previous study by Shirley *et al*[6]. It had three sections. Section A (items1-6) collected information on the demographic data of the participants. Section B (items 7–13) collected information on the working experience of physiotherapists and section C (items 14–20) collected information on knowledge, attitude (role perception and confidence) and counselling practice of physiotherapists in promoting physically active lifestyle in patients as well as barriers to the practice and feasible ways of promoting physical activity to patients (beyond therapeutic exercise).

### Data analysis

Descriptive statistics of frequency, percentages was used to summarise data. Inferential statistics of chi-square was used to determine the association of barriers, work experience, relative physical activity levels of physiotherapists, knowledge and attitude to physical activity promotion with the number of patients counselled in a month.

The results were presented using tables, histograms and pie charts. The level of significance was set at p < 0.05.

## Results

**Figure 1:**
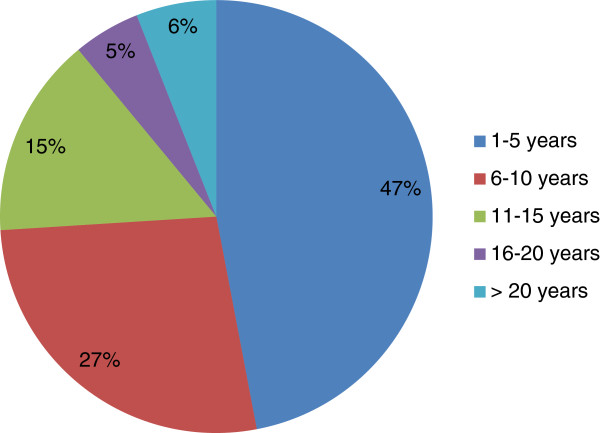
Years of working experience of the Respondents.

A total of 400 questionnaires were distributed and 308 copies were returned, giving a response rate of 77%. 146 (47%) of the respondents had worked for 1 to 5 years while 18 (6%) had a working experience of 20 years and above (Figure 1). The frequency distribution of various states in Nigeria where the questionnaires were distributed is shown in Table 1.

**Table 1:** Frequency distribution of respondents from various States in Nigeria where the Questionnaires were distributed

**STATES**	**Frequency (n)**	**Percentage (%)**
**Lagos State**	94	30.52
**Oyo State**	27	8.77
**Ogun State**	14	4.54
**Osun State**	18	5.84
**Kwara State**	8	2.60
**Enugu State**	22	7.14
**Federal Capital Territory Abuja**	38	12.34
**Jigawa State**	20	6.49
**Edo State**	12	3.90
**Ekiti**	10	3.25
**Imo**	10	3.25
**Plateau**	9	2.92
**Kano**	10	3.25
**Anambra**	16	5.19
**Total**	308	100.00

The mean number of patients seen weekly by physiotherapists was 27.6 ± 18.2 patients. 107 (37%) of the respondents treated 11–20 patients per week while 21 (7.3%) treated more than 40 patients every week (Table 2). 115 (41.1%) of the respondents worked for 31–40 hours per week while 11 (3.9%) of the respondents worked for 50 hours and above per week (Table 2). The mean number of weekly working hours was 33.6 ± 14.7 hours. There were an equal number of the respondents who had undergone post qualification training in physical activity promotion and those who have no post qualification training in physical activity promotion.

**Table 2:** Number of patients seen and working hours per week by the respondents

	**Frequency (n)**	**Percentage (%)**
**Number of patients seen weekly**		
1 – 10	36	12.5
11 – 20	107	37.0
21 – 30	77	26.6
31 – 40	48	16.6
>40	21	7.3
**Total**	**289**	**100.0**
**Working hours per week**		
1 – 10	38	13.6
11 – 20	18	6.4
21 – 30	55	19.6
31 – 40	115	41.1
41 – 50	43	15.4
>50	11	3.9
**Total**	**280**	**100.0**

**Figure 2:**
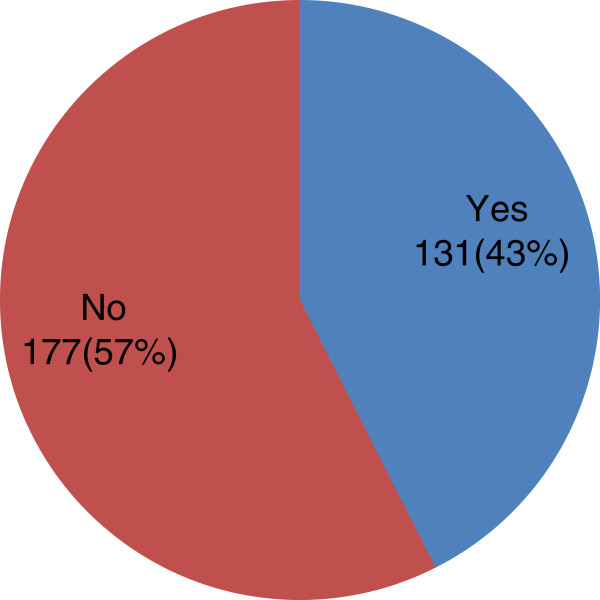
Respondents’ awareness of physical activity recommendation in other countries.

**Figure 3:**
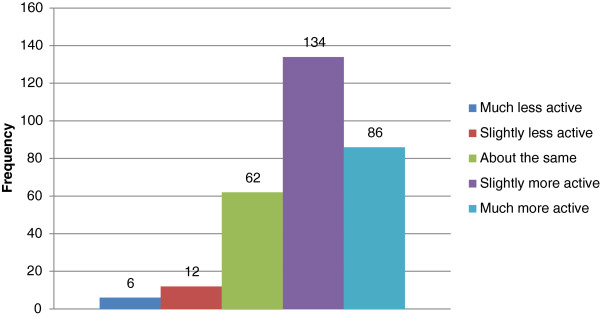
Relative physical activity of respondents compared to other Nigerians of same gender and age.

Concerning the awareness of the physical activity recommendation guideline in any country, 177 (57%) of the respondents had no awareness while 131 (43%) were aware of physical activity recommendation guidelines in other countries (Figure 2). 287 (97%) of the respondents felt that physical activity recommendation guideline is necessary in Nigeria while only 10 (3%) did not feel any need for it. 134 (43.5%) of the respondents considered themselves as slightly more active as other Nigerians while 6 (1.9%) considered themselves as much less active as other Nigerians of same age and gender (Figure 3). 187 (60.7%) of the respondents identified insufficient consultation time as a barrier to physical activity promotion and 294 (95.4%) identified brief counselling integrated into regular consultations as the most feasible means of physical activity promotion in patient management (Table 3).

**Table 3:** Barriers to Physical activity promotion and Respondents’ Views on Feasibility of Different Physical Activity Promotion strategies

**Barriers to promotion**	**Never n (%)**	**Rarely n (%)**	**Sometimes n (%)**	**Often n (%)**	**Very often n (%)**
Insufficient consultation time	52(16.9)	67(21.8)	127(41.2)	32(10.4)	28(9.1)
Lack of counselling skills	150(48.7)	115(37.3)	30(9.7)	7(2.3)	3(1.0)
Lack of remuneration for promoting Physical activity	136(44.2)	73(23.7)	64(20.8)	14(4.5)	15(4.9)
Lack of interest in promoting Physical activity	173(56.2)	72(23.4)	36(11.7)	8(2.6)	17(5.5)
Feeling it would not change the patient’s behaviour	141(45.8)	95(30.8)	56(18.2)	8(2.6)	2(0.6)
Feeling it would not be beneficial for the patient	193(62.7)	81(26.3)	21(6.8)	5(1.6)	4(1.3)
**Feasibility of different physical activity promotion strategies**	**Highly Feasible n (%)**	**Somewhat Feasible n (%)**	**Not sure n (%)**	**Not really feasible n (%)**	**Totally unfeasible n (%)**
Brief counselling integrated into regular consultations	206(66.9)	88(28.6)	8(2.6)	4(1.3)	2(0.6)
Separate one-on-one consultations	81(26.3)	137(44.5)	44(14.3)	35(11.4)	11(3.6)
Group sessions	98(31.8)	134(43.5)	31(10.1)	37(12.0)	8(2.6)
Distribution of resources (eg, brochures)	105(34.1)	120(39.0)	43(14.0)	30(9.7)	9(2.9)

**Figure 4:**
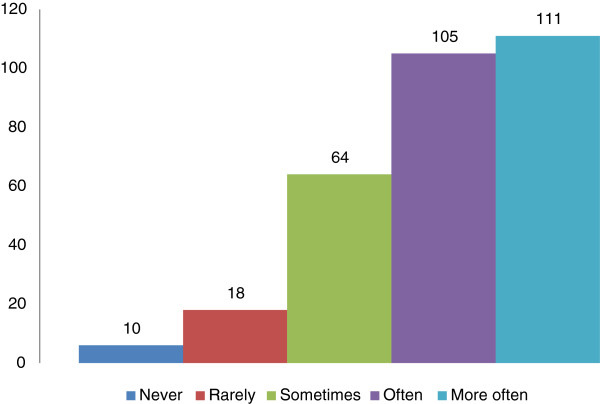
Average number of patients counselled in the last month by the respondents.

One hundred and ninety six (63.6%) of the respondents had high knowledge and two hundred and ninety two (94.8%) had good attitude towards physical activity promotion in patients’ management (Table 4). Only 111 (36%) of the respondents counselled more than 10 patients in the past one month on the benefits of adopting a more physically active lifestyles (Figure 4).

**Table 4:** Respondents Knowledge and Attitude in Physical Activity Promotion

**Variable**	**Strongly Agree n(%)**	**Agree n(%)**	**Not sure n (%)**	**Disagree n(%)**	**Strongly Disagree n(%)**
**Knowledge of Physical activity message**					
Taking the stairs at work and generally being more active each day is enough PA to improve health	64(20.8)	123(39.9)	20(6.5)	91(29.5)	6(1.9)
Half an hour of walking on most days is all the exercise that is needed for good health	31(10.1)	109(35.4)	38(12.3)	102(33.1)	19(6.2)
Exercise that is good for health must make you puff and pant	12(3.9)	48(15.6)	31(10.1)	138(44.8)	74(24.0)
Several short walks of 10 minutes each on most days is better than one round of golf per week for good health	94(30.5)	119(38.6)	58(18.8)	30(9.7)	2(0.6)
**Perception of role**					
Discussing the benefits of a physically active lifestyle with patients is part of the physical therapist’s role	202(65.6)	97(31.5)	5(1.6)	-	3(1.0)
Suggesting to patients ways to increase daily PA is part of the physical therapist’s role	192(62.3)	102(33.1)	10(3.2)	3(1.0)	1(0.3)
**Confidence in promoting Physical activity**					
I feel confident in giving general advice to patients on a physically active lifestyle	186(60.4)	111(36.0)	6(1.9)	4(1.3)	-

PA = Physical activity.

Chi-Square analysis showed that there was no significant association between the knowledge of respondents in physical activity promotion with their counselling practice to patients in a month (Table 5). Also there was no significant association between the attitude (role perception and confidence) of respondents to physical activity promotion with their counselling practice to patients in a month (Table 5).

**Table 5:** Relationship between knowledge and attitude (role perception and confidence) in physical activity promotion and number of patients counselled in a month

**Variable**		**Counselled <10 patients/Month (n = 197) n (%)**	**Counselled ≥10 patients/Month (n = 111) n (%)**	ℵ^2^	**p**
**Knowledge of Physical activity message**					
Taking the stairs at work and generally being more active each day is enough PA to improve health	High	119(63.3)	69(36.7)	0.01	0.547
	Low	76(63.3)	44(36.7)		
Half an hour of walking on most days is all the exercise that is needed for good health	High	90(64.3)	50(35.7)	0.81	0.419
	Low	105(62.5)	63(37.5)		
Exercise that is good for health must make you puff and pant	High	44(72.1)	17(27.9)	2.55	0.072
	Low	151(61.1)	96(38.9)		
Several short walks of 10 minutes each on most days is better than one round of golf per week for good health	High	183(61.4)	68(35.2)	0.03	0.532
	Low	52(53.6)	45(46.4)		
**Perception of role**					
Discussing the benefits of a physically active lifestyle with patients is part of the physical therapist’s role	High	189(63.2)	110(37.6)	0.05	0.567
	Low	6(66.7)	3(33.3)		
Suggesting to patients ways to increase daily PA is part of the physical therapist’s role	High	184(62.8)	106(37.2)	0.68	0.297
	Low	11(73.3)	4(26.7)		
**Confidence in promoting Physical activity**					
I feel confident in giving general advice to patients on a physically active lifestyle	High	186(62.4)	112(37.6)	3.17	0.067
	Low	9(90.0)	1(10.0)		
I feel confident in suggesting specific PA programs for my patients	High	182(62.5)	109(37.5)	1.34	0.186
	Low	13(76.5)	4(23.5)		
Physical therapists should be physically active to act as a role model for their patients	High	180(62.5)	108(37.5)	1.26	0.190
	Low	15(75.0)	5(25.0)		

**P**-level of significance set at < 0.05, PA = Physical activity.

Chi-square analysis showed that there was a statistically significant association between low practice of physical activity promotion in patient management and insufficient consultation time (ℵ^2^ = 3.36, p = 0.043) (Table 6). There was also a significant association between practice of physical activity promotion in patient management and years of working experience (1–5 years) of physiotherapists (ℵ^2^ = 11.37, p = 0.023) as well as the relative physical activity levels of physiotherapists (ℵ^2^ = 11.82, p = 0.037) (Table 7).

**Table 6:** Relationship between barriers to physical activity promotion and the number of patients counselled in a month

**Barriers to promotion**		**Counselled <10 patients/Month (n = 197) n (%)**	**Counselled ≥10 patients/Month (n = 111) n (%)**	ℵ^2^	**P**
Insufficient consultation time	High	71(59.7)	48(40.3)	3.36	0.043*
	Low	124(65.6)	64(34.4)		
Lack of counselling skills	High	163(61.5)	102(38.5)	2.65	0.070
	Low	30(74.4)	11(25.6)		
Lack of remuneration for promoting PA	High	133(63.6)	76(36.4)	0.03	0.480
	Low	62(62.6)	37(37.4)		
Lack of interest in promoting PA	High	160(65.3)	85(34.7)	2.05	0.100
	Low	35(55.6)	28(44.4)		
Feeling it would not change the patient’s behaviour	High	145(62.0)	89(38.0)	0.76	0.233
	Low	50(67.6)	20(32.4)		
Feeling it would not be beneficial for the patient	High	173(62.5)	104(37.5)	0.87	0.233
	Low	22(71.0)	9(29.0)		

**P**-level of significance set at < 0.05, * = significant.

**Table 7:** Relationship between work experience, relative physical activity levels of physiotherapists and the number of patients counselled in a month

**Variable**	**Counselled <10 patients/Month (n = 195)**	**Counselled ≥10 patients/Month (n = 111)**	ℵ^2^	**p**
**Work experience (years)**				
1 – 5	106	40	11.37	0.023*
6 – 10	48	34		
11 – 15	25	21		
16 – 20	10	6		
>20	6	9		
**Relative physical activity levels**				
Much less active	4	2		
Slightly less active	5	7		
About the same	41	21		
Slightly much active	94	40		
Much more active	44	42	11.82	0.037*

**P**-level of significance set at < 0.05, * = significant.

## Discussion

The aim of this study was to determine the knowledge, attitude and practice of physiotherapists towards promotion of non-treatment physical activity for better health in patient management.

It was observed that about two thirds of the respondents had a high knowledge of physical activity promotion. Almost all the respondents had very good attitude (role perception and confidence) towards physical activity promotion. However, only one third of the respondents counselled more than 10 patients in a month. It was also observed that 60.7% of the respondents identified insufficient consultation time as a barrier to the promotion of active lifestyle among patients. Almost all the respondents identified brief counselling integrated into regular consultations as the most feasible means of physical activity promotion in patient management. Most of the respondents considered themselves as more physically active than other Nigerians of the same age and gender. More than half of the respondents were not aware of physical activity recommendation guidelines in other countries while almost all of them felt that physical activity recommendation guideline is necessary in Nigeria. The main limitation of this study was the relative small sample size.

The observation that two thirds of the surveyed physiotherapists in Nigeria had high knowledge of physical activity promotion yet most of them counselled less than 10 patients in a month implies that Nigerian physiotherapists at present operate mainly in the tertiary prevention capacity. A similar study by Shirley *et al.*[6] observed that more than half of the surveyed Australian physiotherapists (54%) counselled 10 or more patients to lead a more physically active lifestyle (beyond therapeutic exercises) in a month. They also observed that physical therapists who gave patients more physical activity life style advice appeared to have greater knowledge about physical activity promotion. This means that 18% more physiotherapists in Australia practice physical activity promotion in patient management than those in Nigeria. The reason may be that there are fewer physiotherapists in Nigeria with large number of patients to manage than in Australia. As such they will be constrained by time to counsel their patients on having a more physically active lifestyle. Therefore, it is not surprising that 60.7% of the surveyed physiotherapists in Nigeria identified insufficient consultation time as a barrier to the promotion of physically active lifestyle among patients.

There was no significant association between the attitude of the surveyed physiotherapists towards physical activity promotion in patient management and their counselling practice. This means that the good attitude of almost all surveyed physiotherapists in Nigeria towards physical activity promotion had no influence on their counselling practice to patients. This also may be attributed to lack of time as few physiotherapists are available for large number of patients. Shirley *et al.*[6] reported that almost all the surveyed Australian physiotherapists had very good role perception and confidence in promoting physical activity to patients and this influenced their practice of it. In 3 states in USA, about 54% of physical therapists believed that it was part of their role to be involved in promotion of health and fitness [12]. Buffart *et al.*[13] reported that 98% of physicians that participated in their study believed that physical activity promotion was part of the physician’s role but fewer physicians felt confident in giving specific physical activity advice to patients. Leijon *et al.*[14] reported that among the various health care professionals in Sweden, physical therapists provided the highest number of physical activity referrals and physicians provided the lowest. This difference may be due to the fact that physiotherapists have extensive training on exercise prescription for both primary and tertiary prevention of diseases and disabilities.

There was a significant association between low practice of physical activity promotion in patient management and insufficient consultation time. This suggests that low practice of physical activity promotion in patient management observed in this study is mainly due to insufficient consultation time. Bull *et al.*[15] and van der Ploeg *et al.*[8] reported that lack of time was a major barrier to physical activity promotion in the clinical setting.

The finding that almost all the surveyed physiotherapists in this study identified brief counselling integrated into regular consultations as the most feasible means of physical activity promotion in patient management may also be due to insufficient consultation time. Shirley *et al.*[6] reported that physical therapists indicated that separate, one-on-one consultations are less feasible for physical activity promotion but incorporating non-treatment physical activity advice into normal consultations is deemed feasible by almost all. However, van der Ploeg *et al.*[16] and van der Ploeg *et al.*[8] reported that a physical activity counsellor can effectively improve patients’ daily physical activity levels in a series of one-on-one counselling sessions.

There was a significant association between practice of physical activity promotion in patient management and years of working experience of the respondents suggesting that those who had 1–5 years’ work experience counselled more patients. The reason for this may be that a larger number of the surveyed physiotherapists were in this group.

There was also a significant association between practice of physical activity promotion in patient management and the relative physical activity levels of the surveyed physiotherapists. This means that the physiotherapists in Nigeria who considered themselves much more physically active than other Nigerians of the same age and gender counselled more patients on the importance of adopting a more physically active lifestyle.

Although majority of the physiotherapists in Nigeria (97%) saw the necessity for physical activity recommendation guideline in Nigeria only 43% were aware of physical activity recommendation guideline in other countries. Shirley *et al.*[6] reported that only one third of the surveyed physical therapists were aware of the national physical activity guidelines.

### Study limitations

The questionnaire used in collecting data in this study was not pretested and validated for the Nigerian environment. This is viewed as a limitation, although the contents in the questionnaire was well understood by the respondents and matched the work environment in Nigeria.

The sampling technique also proved to be a limitation because physiotherapists from only fourteen states of the country participated in the study. The selected states were those that had relatively higher number of currently practicing physiotherapists and represented almost all the 6 geopolitical zones of Nigeria. We could therefore infer that the results were representative of the physiotherapists in the country.

## Conclusion

Physiotherapists in Nigeria have good knowledge and good attitude towards promotion of physically active lifestyle in their patients but do not counsel many of them. Insufficient consultation time was the main factor that influenced the practice. They believed that integrating brief counselling into usual treatment sessions is the most feasible form of physical activity promotion in patient management.

Based on the findings of this study, it is hereby recommended that physiotherapists in Nigeria should create more time for consultation in order to integrate brief counselling of patients on the importance of leading a more physically active lifestyle into usual treatment sessions.

Health policy makers, Clinic managers and Heads of Physiotherapy Clinics in Nigeria who are in position to determine how much time physiotherapists could spend with patients, should also be advised on planning the clinics in such a way that the consultation time spent with patients would be appreciable enough to accommodate counselling patients on the importance of leading more physically active lives.

The capacity of Nigerian physiotherapists to promote non-treatment physical activity among their patients can be improved further especially by emphasizing it in physiotherapy curriculum. There is also the need for physical activity recommendation guideline in Nigeria.

## Competing interests

The authors declare that they have no competing interests.

## Authors’ contributions

HAA conceptualized the study, participated in the design of the methodology and drafted the manuscript. CNO was involved in data acquisition, analysis and data interpretation. OAF participated in the design of the study’s methodology, conducted the analysis, interpretation of data and prepared the manuscript for final publication. OAO developed the study’s methodology and reviewed the manuscript for important intellectual content. All authors read and approved the final manuscript.

## Pre-publication history

The pre-publication history for this paper can be accessed here:

http://www.biomedcentral.com/1472-6963/13/21/prepub
